# Towards functional precision medicine? Evidence standards of organoids as patient-specific models

**DOI:** 10.1007/s40656-026-00720-x

**Published:** 2026-03-02

**Authors:** Sara Green, Henrik Vogt, Maxence Gaillard

**Affiliations:** 1 Section for History and Philosophy of Science, Department of Science Education, University of Copenhagen, Copenhagen, Denmark; 2 Department of Public Health, Centre for Medical Science and Technology Studies, University of Copenhagen, Copenhagen, Denmark; 3 Department of Community Medicine and Global Health, Faculty of Medicine, Institute for Health and Society, University of Oslo, Oslo, Norway; 4 Centre for Medical Ethics, Institute of Health and Society, University of Oslo, Oslo, Norway

**Keywords:** Functional precision medicine, Evidence standards, Patient-derived organoids, Cancer medicine, Cystic fibrosis

## Abstract

Evidence-based medicine (EBM), with meta-analysis of randomized clinical trials as its gold standard, has been criticized for failing to represent the individuality and variability of disease. Precision medicine (PM) has been proposed as an alternative to EBM’s “averaging approach”, leveraging genomic and other biological information at the individual level. However, PM is still an emerging and changing concept. It is unclear what constitutes acceptable evidence, when the number of patients with a specific condition approaches one. Despite large investments, PM´s overall capacity to predict and improve treatment responses remains limited. This raises the question of whether PM has failed, or whether another strategy can improve the situation. Here, we examine the implications of *functional precision medicine* (FPM), a strategy aiming to bridge the gap between genomic information and phenotypic complexity through functional testing of treatments on patient-derived organoid (PDO), an advanced form of cell culture. We unpack how observed treatment effects in such personalized models are emerging as a means to predict treatment efficacy in individual patients. Drawing on exploratory interviews with scientists at the forefront of clinical implementation, we examine the philosophical implications of FPM in the contexts of cystic fibrosis and cancer. We unpack how the “functional approach” addresses biological complexity by black boxing many mechanistic details and focusing on phenotypic responses in PDOs. Moreover, we show that, to work as personalized models, they paradoxically must be validated by developing the same type of population-based evidence they aim to reduce reliance on.

## Introduction

Evidence-based medicine (EBM) has been criticized for implying a universalist epistemology that tends to accept averages of treatment effects in populations as adequate knowledge for decisions about individuals, failing to account for variation between patients (Deaton & Cartwright, 2018; see also Stegenga, 2018a, b). Addressing such shortcomings, proponents of precision medicine (PM), also known as personalized medicine, stress the need to identify how molecular biomarkers, e.g., genetic mutations, or the interplay between them, impact disease development and can serve as predictors of treatment response at the individual level (Huang & Hood, 2019).^1^ The PM vision thus includes not only a promise of disrupting clinical management, but also an underlying epistemological promise of enhancing EBM itself and radically improving our ways of knowing what is useful at the individual level (Beckmann & Lew, 2016). At present, however, PM is still an emerging and evolving concept, and it is not yet clear what it will look like as an alternative evidence-basis for a *scientific*, yet *individualized*, approach, or how to best combine different forms of evidence (Woodcock & Marks, 2019; Fuller et al., 2024; Vogt, 2025). This paper explores what constitutes reliable evidence of effectiveness in PM from the perspective of what Fuller (2025, p. 410) calls “radical personalized medicine”, i.e., where the aim is to use a *patient-specific* organoid model to make predictions about an individual. This approach is currently promoted as “*functional precision medicine*”. We explore the following questions: How are patient-specific models envisioned to predict what works in the individual case, and what are the philosophical and scientific implications of FPM? What do practicing scientists view as acceptable evidence basis for personalized treatment allocation in this context, and what are the current potentials and challenges?

PM rekindles long-standing debates in philosophy of science and medicine about the relative strength of statistical evidence versus mechanistic knowledge about causal difference-makers (Russo & Williamson, 2007; Parkkinen et al., 2018; Stegenga, 2022). Diseases and patients are increasingly stratified into subgroups based on molecular variations, which reduces the number of patients (n) with a given disease, ultimately converging toward one individual (Schork, 2015; Wadmann & Hauge, 2021). A departure from reliance on population-based methods has been considered justifiable, if the limitations to trial power (fewer patients) can be outweighed by (i) a more comprehensive mechanistic understanding of disease causes and treatment effects, and (ii) larger effect sizes of targeted treatments (Tonelli & Shirts, 2017; Andreoletti, 2018; Tonelli & Williamson, 2020). Neither of these conditions are currently satisfied, at least for complex diseases such as cancer (Plutynski, 2022; Tabery, 2023). Hence, some scholars have warned that falling for lofty high tech promises of PM and prioritizing expert opinion and mechanistic reasoning about what individuals need over population-based evidence could “open the flood-gates to poorly founded medicine” (Vogt & Hofmann, 2022; see also Kane et al., 2021). For example, circumventing the requirement of RCTs may reduce standards of accountability and transparency for industrial benefits and result in drug approval without sufficient evidence (Stegenga, 2018a, p. 157). Thus, PM is faced with an urgent need to bridge the knowledge gap between “omics” information and what works at the phenotypic (functional) level of individual patients, i.e., experienced and measured treatment effect.

Functional precision medicine (FPM) promises to address this challenge by using material in vitro models of individual disease, acting as stand ins for the patient. The FPM concept was coined by Letai in 2017 in a *Nature Medicine* article on precision oncology, stressing the need to move PM beyond genomics (Letai, 2017). The concept has been taken up in several scientific papers (e.g., Flobak et al., 2022; Acanda De La Rocha et al. 2024), and a Society of Functional Precision Medicine has been founded.^2^ FPM is premised on the idea that harvested cells grown in a lab as *organoids* can work as a patient-specific in vitro model of that patient’s disease. Organoids are advanced in vitro cell culture models that mimic the structure and function of organs or tumors (Caianiello et al., 2023). The use of 3D cell cultures in biological research is far from new (Simian & Bissel 2017), but they have regained new attention in PM due to the potential of serving as *patient-specific* models. For example, patient-derived organoids (PDOs) based on tumor biopsies are hoped to represent the tumor of that very patient to an extent where the model can predict treatment efficacy at the individual level (Green et al., 2022).

The vision of FPM is to use these in vitro model to perform *functional tests*. What functional means here is that one uses the “functional response” of the model to a candidate treatment. In other words, the FPM vision is to make the dynamic change in the model a predictor of treatment response in the patient, instead of relying solely on “static” biomarkers, or sets thereof, that were found in the patient before the treatment was tried. Results in such models are thought to be more directly related to functions at the phenotypic level (Letai et al., 2022). In a sense, the long-standing tradition among clinicians of simply trying a treatment out in a patient to see if it works “functionally” in that individual, is a form of FPM. In this novel approach however, the idea is to first try it out on a stand-in (the PDO). Considering the direct *material* relationship between the in vitro model and the in vivo patient, Walker et al. (2019) have suggested that PDOs present a new kind of evidence that is neither based solely on mechanistic insights nor on statistical inferences from large samples. In 2019, Walker and colleagues wrote that the “exact form of tissue cultures that could be suitable for the purpose of treatment regimen personalization has not yet been determined” (Walker et al., 2019, p. 107). But as PDO models are now beginning to move into clinical spaces, it is time to reexamine the evidence status and clinical utility of PDOs.

This paper is intended as a philosophical analysis on evidence standards in organoid research, combining literature analysis and insights from exploratory interviews with practicing scientists working at the clinical frontiers of application of PDOs. Drawing on an analogy made by our informants between PDOs and antibiograms in clinical microbiology, we unpack how FPM is thought to pave the way for “molecular-agnostic predictions” (Betge & Jackstadt, 2023). At the same time, our analysis also reveals that PDOs do not straightforwardly provide evidence about individuals. Rather, evidence requirements depend on the clinical situation of target patients, the biological complexity of the studied disease, and the expected effect sizes of targeted treatments. Our analysis also exposes how validation of PDOs requires clinical trials that document population-level benefits, thus relying on similar evidence as the field of FPM is hoping to reduce the need for. Rather than a radical departure from EBM, FPM in the examined contexts combines individualized and population-level approaches.

## Materials, methods, and structure of the paper

This exploration of what constitutes evidence in FPM is motivated by questions emerging from a previous study, where we mapped the types and status of translational organoid research based on a review of published philosophical and scientific literature on PDOs, as well as information provided on registered clinical trials including PDOs (Vogt et al., 2025). This study revealed that the areas where PDOs are currently the closest to clinical implementation are cystic fibrosis (CF) and cancer. In this paper, we draw on the previous literature analysis as well as semi-structured exploratory interviews with scientists to provide some insights into the implications of initial attempts to implement PDOs in clinical practice. Our informants are all working at the forefront of clinical implementation of PDOs in the two contexts. Our primary aim was to explore how these pioneers view the future of the field and to get insights into what they experience as potentials and barriers to clinical translation, with special focus on what evidence is required for wider clinical adoption of PDO-based treatment decisions.

As candidate informants for our study, we identified scientists who have reported, in significant publications, efforts to clinically implement PDOs to guide treatment decisions for patients with cancer or cystic fibrosis. The researchers identified were also all members of institutes, departments, or research groups involved in ongoing clinical trials or studies with patients.^3^ Some of these researchers were also active in public communication and promotion of the vision of using PDOs in the clinic, authors of opinion papers, and review articles. Within this context, we strived to respect a balance in terms of researchers’ field of specialization, geographical location, and gender and sent invitations to potential informants via email.^4^

Ten online semi-structured interviews lasting up to 60 min, mostly with senior researchers and group leaders, were conducted between September 2022 and January 2023. The study was given ethical approval by the Ethical Council Board of the Norwegian Centre for Research. The interviewees provided informed consent to participate in the interviews. The interviewees were not approached as bearer of specific projects (as laboratory leaders) but in their capacity as to offer unique insider-perspectives on the status and future of the whole field. The interview guide hence contained questions about the prospects and challenges for clinical implementation in general, as well as questions relating specifically to their individual projects. The interviews were transcribed ad verbatim, then anonymized the text by removing identifying or confidential details. In analyzing the interviews, we used an abductive analytical approach, where analytic ideas and codes (e.g., barriers to clinical implementation, perceived evidence requirements) were developed in an iterative process (Timmermans & Tavory, 2012). Once major themes were identified, the analysis was also informed by our own reading of scientific articles in the field, including reviews and papers presenting evidence of using PDOs in clinical settings. We were also interested in the scientists’ use of metaphors and analogies in describing the use of PDOs, including “avatar models” and the “antibiogram” discussed below.

The paper is structured as follows. We first introduce how organoids are envisioned to function as “patient avatars” to guide treatment selection in the contexts of oncology and CF (Sect. 3). We unpack an analogy, revealed through our interviews, between the new PDO models and antibiograms used in clinical microbiology (Sect. 4). We then elaborate on what, according to our informants, it would take to validate PDOs as predictive clinical models in the contexts of cystic fibrosis and cancer (Sect. 5). We end with reflections on the evidence status of PDOs as “personalized” models in FPM (Sects. 6 and 7).

## Organoids as “patient avatars” in cystic fibrosis and cancer medicine

In recent years, organoids have been developed to mimic a variety of different tissue types, such as liver, brain, kidney, pancreas, lung, heart, and the retina, and they are also used to model disease processes. More recently, organoids have been developed towards the aim of realizing “personalized” testing in PM (Green et al., 2022; Dam & Green, 2022; Hinterberger & Bea, 2023). In line with this vision, our informants presented organoids via metaphors such as “patient avatars” (senior researchers from both industry and academia), “patients in a dish” (senior researcher, industry), “individual predictors”, and “living biomarkers” (senior researcher, academic, cystic fibrosis). In the following, we unpack what they mean by such terms and why such models are needed in the research on and clinical treatment of cystic fibrosis and cancer.

Cystic fibrosis (CF) is one of the most common life-shortening genetic diseases, caused by mutations in the transmembrane conductance regulator (CFTR) gene. The mutational lesions disrupt central functions of the CFTR protein, such as transportation and regulation of chloride ions and water across cell membranes. A defect in this gene leads to changes in mucus, sweat and digestive juices, causing digestive and lung-related problems. CFTR-modulating drugs that target the CFTR receptor, such as ivacaftor, have become available. The snag is that, despite being a monogenetic disease, CF can be caused by up to 2,000 different mutations (and counting) in the same gene, and each can result in a different, unpredictable response to available treatments (Hillenaar et al., 2022). This provides a structural problem for drug approval for patients with diverse mutations, as there are often too few patients with specific mutations or combinations to make clinical trials possible to study the effect of CFTR-modulating drugs for just that mutation, at least in the short term (Arora et al., 2021). As a publication suggesting use of the PDO-based strategy put it: “The current standard for clinical trials, the randomized controlled trial (RCT), requires a large homogenous population to predict drug efficacy. Due to the number of known mutations in CFTR, it is impossible to conduct these RCTs on ivacaftor treatment for every possible mutation” (Ogden et al., 2021, p. 7).

To close this gap, the authors propose a specific form of functional organoid test called the forskolin-induced swelling (FIS) assay (Ogden et al. 2021). The FIS assay was introduced in 2013 and uses as a functional readout the capacity of intestinal cells to swell after exposure to forskolin (Dekkers et al., 2013a, 2013b; Ikpa et al., 2014).^5^ Normal cells expand through the uptake of liquid, whereas cells from CF patients collapse and do not swell. Hence, cell swelling is used as a proxy for normal functioning CFTR proteins. Yet, if pre-incubated with a CFTR-modulating drug, the gene function in organoids from CF-patients can be therapeutically restored, allowing the intestinal organoids from CF patients to swell like normal cells. Combining forskolin- and CFTR-modulating drugs in a swelling assay with PDOs, developed from specific CF patients’ cells, can thus help identify potentially beneficial treatments that work for the specific mutations of individual patients (Mou et al., 2015). To link this to the FPM concept: The predictor here is the functional, dynamic change indicating whether the drug will work in the patient – the degree of swelling in the organoid rather that a “static” DNA mutation. A negative test result (no response to treatment) is also clinically relevant, as this could reduce overtreatment with associated side effects and unnecessary additional costs (de Poel et al., 2020).

A similar challenge is faced in oncology, although cancer is a much more common disease than CF. Advances in genomic analysis have revealed that mutations and response rates vary greatly between cancer patients, even if they have the same overall diagnoses. An increasing number of mutations have been associated with resistance to standard treatments, and drugs targeting specific mutations have been developed. However, knowing whether *specific* patients will benefit from specific treatments remains a persistent challenge (Dam et al., 2022; Plutynski, 2022). One of our informants explained that cancer cells can have thousands of different mutations, yet the relevance of most of these mutations is unknown. Testing treatment directly on patients is the only way to be sure about the effect. But as this might expose patients to adverse side-effects with unclear benefits, an intermediate patient-specific model is considered needed. In the words of one of our informants:

Cancer would be an ideal disease for the same approach [as CF]. […] Doctors always take a small part of the tumor out for diagnosis, you need very little, and you can grow cancer organoids and do exactly the same thing that was done for cystic fibrosis. [Y]ou expose the line of organoids you made from the patient to all the available drugs or drug combinations and then pick the best one. (senior researcher, industry)

**Fig. 1:**
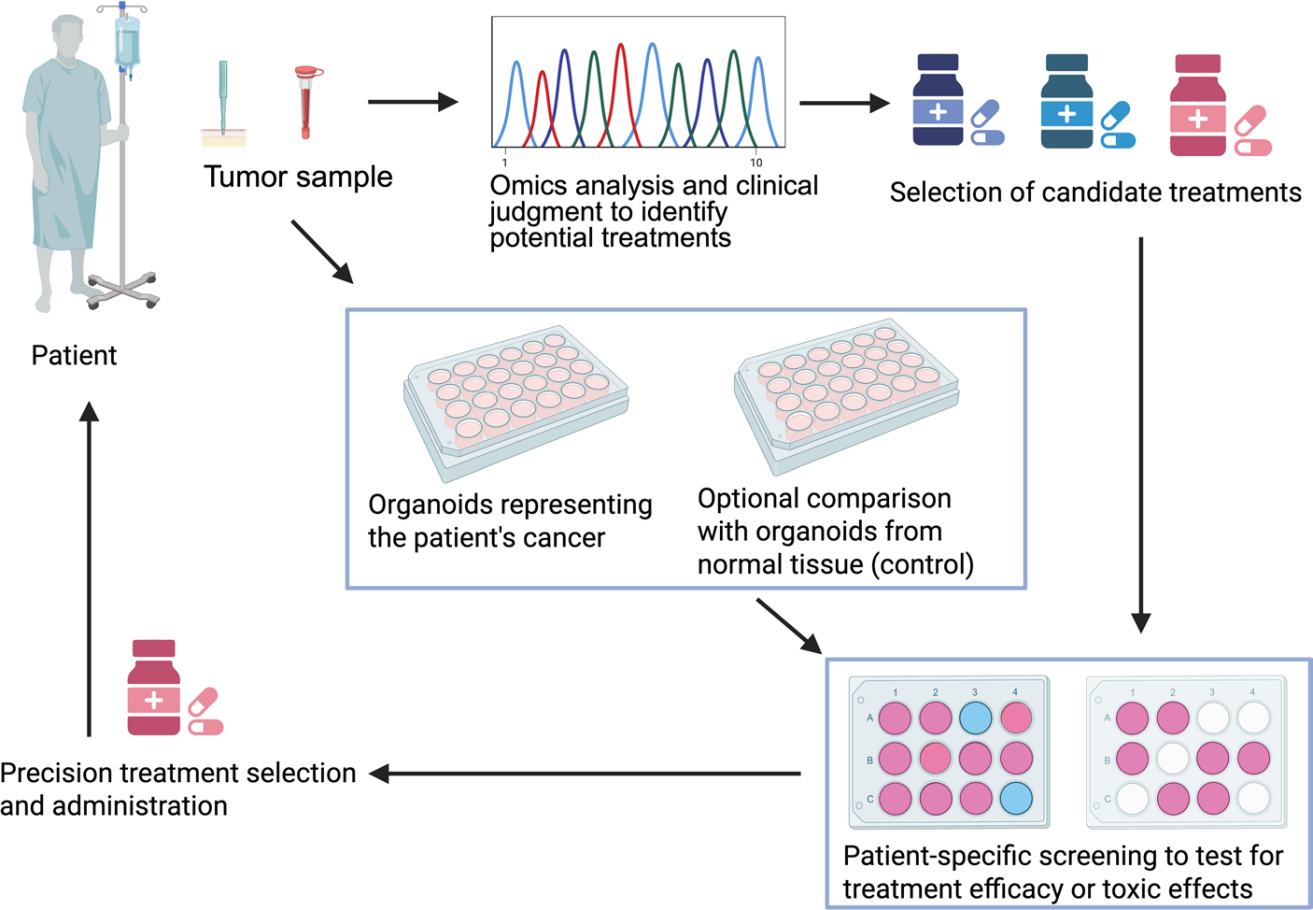
The use of PDOs to test for effective treatments at the individual patient level. See the main text for details. The figure was created by the authors via BioRender.

The described use of PDOs for patient-specific drug screening is illustrated in Fig. 1. The first step is to derive cells from the individual patient on the basis of a biopsy, e.g., from a tumor in the case of cancer or intestinal cells in the case of CF. A small part of the sample is typically used for genome sequencing to identify potential targeted treatments (and in the case of cancer also to validate that the biopsy is from the tumor, and not normal cells). The remaining biopsy material can then be grown as organoids for drug screening in comparison to an untreated control cultured under the same laboratory conditions. In the ideal setting, based on the *functional response* of the PDO models (i.e., swelling of cells from CF patients or reduced viability of tumor organoids), a PDO-based recommendation can be presented to the treating physician for individualized decision-making.

CF and cancer are thus important areas for realizing the vision of using PDOs as “patient avatars”, because it is not possible to predict treatment response from genome sequencing alone and because there are too few patients with a specific disease to conduct traditional clinical trials. However, while PDOs may appear to offer a straightforward way of testing treatment efficacy “directly” on the patient’s “avatar”, the following shows how their evidence status is still associated with uncertainties. We next turn to how researchers at the forefront of clinical implementation view the status of PDOs as evidence in FPM.

## PDOs – a new form of medical evidence?

Genomic precision is often considered as a hallmark of precision medicine. Yet, the promotion of organoids as the “Method of the Year 2017” by *Nature* also reflects an increasing awareness that understanding and predicting living systems requires studying and intervening on life in three dimensions (Nature editorial 2018). Interestingly, the editorial depicts organoids somewhat ambiguously as a “shiny new tool” and as a “refurbished” one (Nature editorial 2018). The novelty is not the use of 3D cell cultures as such, but lies in the convergence of laboratory methods to grow human tissue, knowledge of tissue and disease development, and insights from omics approaches. These things combined have raised hopes for a groundbreaking “possibility of a science of the individual, since the tumor cancer cell population actually is an individual (patient) in vitro” (Boniolo, 2017, p. 29). We were therefore surprised to learn how our informants view PDOs in analogy to a much older approach in medicine, namely the antibiogram in clinical microbiology.

An *antibiogram* is a laboratory report outlining information about the susceptibility and resistance of infectious bacteria to antibiotics (Truong et al., 2021). Shortly after the introduction of penicillin in the early 1940s, it became evident that bacterial strains displayed variation in response to antibacterial agents, with some being susceptible and others being resistant. Standardized antibiotic sensitivity tests were later developed and adopted by national health agencies for infection control measures in and across hospitals (Reller et al., 2009). In some cases, e.g., if a patient has chronic infections, a functional tests of antibiotic susceptibility on cells from the patient’s blood, urine, or wound swaps can help identify the best treatment plan (Corbin et al., 2022). Clarifying the connection between the individualized antibiogram and PDO-based testing, two informants said the following:

We would use the patient derived organoids to either anticipate or nominate new drug therapies that a patient should have or could have. […] This is exactly the same thing that you’d expect if you had a culture taken off your throat – you get a sore throat and they’re asking question what antibiotic you would use or – you know, other sites where we take regularly take cultures. (senior researcher, academic, cancer)

This is very comparable to what we do with bacterial infections, where, for the past 60 years, when you have a bacterial infection to your lung, you grow the bacteria in the lab, expose them to all available antibiotics, pick the one that kills the bacterium, and this is the one that you give to the patient. (senior researcher, industry)

Because PDOs, like personalized antibiograms, are based on cells from a specific patient, they are envisioned stand-ins for individual patients as a kind of “avatar” or “surrogate model” (see also Bolker, 2009; Green et al., 2021). Importantly, however, the inference from the in vitro test to the best treatment option for the patient is not based on a solid mechanistic account of the model, i.e., of *why* a drug works or does not work:

[An organoid] is the best surrogate of the tumor, but that doesn’t mean you understand it… Here we call our test the “chemogram”, as compared to the antibiogram, because when you go to a diagnostic lab, because, say, you have a pathogenic infection of some sort, a bacterial infection, they are not going to try to find which bacteria is the pathogen. They’re going just to try to identify which antibiotic works for you. (senior researcher, academic, cancer)

The analogy between PDO-based treatment screening and antibiograms is philosophically intriguing for several reasons. Etymologically speaking, the notion of a *-gram* comes from the ancient Greek *gramma* (something written, drawn, or otherwise documented) and the related activity, *grafein* (to write out).^6^ We interpret the notion of a “chemogram” in the latter quote as an approach that lets the patient’s cell “write out” the possible chemical cures through a functional signal. Like the antibiogram, this interpretation does not rest on a comprehensive genomic mapping or mechanistic understanding of the causal pathways of disease development and treatment efficacy. Indeed, since one does not really need detailed molecular information to make clinically relevant predictions via such an approach, this approach has also been called “*molecular-agnostic predictions*” (Betge & Jackstadt, 2023).

Being agnostic about many molecular mechanisms does not make organoid research distinct from, nor opposed to, genetic and mechanistic research. As Walker et al. (2019) emphasize, the perceived need for an in vitro model that shares the individual’s genetic make-up *is* a mechanistic rationale committed to the importance of causal difference-makers at the molecular level. PDOs also provide important models for mechanistic research, e.g., in studies on evolutionary pathways of treatment resistance in oncology (Chai et al., 2023; Seidlitz et al., 2022). However, given the number of mutations and complexity of causal pathways underlying both CF and cancer, deriving reliable predictions for clinical decision-making based on detailed mechanistic modeling has – at least so far – not been successful. Whereas some organoid researchers view PDOs as stepping-stones for linking traditional biomarkers to patient-responses, e.g., through high-throughput genomics and AI,^7^ to arrive at bottom-up predictive models, others view PDOs as a less reductionistic strategy. By representing treatment response at the level of cell cultures or (mini) tissues, the use of PDOs as “living biomarkers” serves as a bridge between the genetic and phenotypic level, which is more practically feasible for arriving at a clinically useful result. In this context, predicting *that* a treatment works is more important than explaining *why*. As expressed by an informant:

I really feel that [difference], as a researcher, because the other part of my lab is really into, you know, molecular mechanisms, signaling pathways, even biophysics, like: How do things work? What’s the causality? Here, we don’t need to understand [that]. That means, we do not understand *why* and *how* this tumor is different from the other patients; What’s the oncogenic driver? What’s the specificities? Nothing, we do not try to understand anything, we just want to find the best drug for this tumor. (senior researcher, academic, cancer)

Given this clinical aim, PDO models provide a more practically feasible “epistemic shortcut” (Walker et al., 2019, p. 109) through the complexity of factors influencing treatment response. Regardless of the potential future success of research focused on “static” genetic biomarkers and molecular mechanisms, FPM may thus allow for faster implementation of personalized treatment selection. In the words of a senior cancer researcher:

I think that the area of functional precision medicine is really where we should go. We don’t need to understand everything about the tumor to be able to provide the right treatment. We know that, for most cancers in oncology, there are very powerful therapeutic options, but it’s still very difficult to understand which are the patients that would benefit from these very expensive treatments. (senior researcher, academic, cancer)

As expressed in this quote, an FPM approach may also help stratify the patient population to select the right patient to receive available (and expensive) precision treatments (see also Wadmann & Hauge, 2021; Dam et al., 2022). The scope of parameters by which patients vary is large, but PDOs can reduce the dimensionality of this complexity to a *functional* readout in a simple in vitro system.

In summary, PDOs are envisioned as patient-specific models for functional testing of treatment efficacy for individual patients, specifically in the contexts of CF and cancer. Our informants emphasize that the evidence status of PDOs does not stem from a bottom-up understanding of disease-related mechanisms. Rather, PDOs are understood as functional tools that allow them to ignore the complexity of molecular interactions, analogous to antibiograms in clinical microbiology. Nevertheless, the predictability of PDOs cannot be taken for granted. We therefore next turn to the question of what it takes to validate PDOs for clinical use.

## Validation of the clinical relevance of PDOs

The validity of PDOs as reliable predictors of individual treatment response has been explored in retrospective and prospective observational studies, also called “co-clinical trials” (Aberle et al., 2018).^8^ In such studies, the readout of PDOs is compared to the disease status and treatment response in the same patients. The typical design of such observational studies was explained by an informant:

What people tend to do is to describe a particular clinical context, for instance a phase 1–2 clinical trial. Start with patients, document the patient [responses], but at the same time take a sample from the tumor of the patient, if we talk about cancer, perform your phase 1–2 clinical trial. In parallel, perform your organoid assay, and then compare the outcomes. So, what the organoid assay may predict and what happened in the clinic. That’s typically what all these studies do. (senior researcher, industry)

Some observational, co-clinical trials have already documented promising results for CF (e.g., Ramalho et al., 2021; Muilwijk et al., 2022) and some types of cancer, e.g., colon, pancreatic, and gastric cancer (Ooft et al., 2019; Beutel et al., 2021; Seppälä et al., 2022; Schmäche et al., 2024). However, according to our informants, stronger evidence is needed to fully assess and document the *clinical utility* of using PDOs in clinical decision making. Whereas evidence is growing the PDOs are valid markers of expected treatment response, it must also be documented that using PDOs can *improve patient outcomes* by facilitating better treatment decisions. Given the positive results from observational trials, several informants contended that the field is now ready to test the clinical benefits in interventionist clinical trials:

I think we’ve had too many studies that were retrospective […]. I think it’s nice as a proof of concept, very early phase development of organoids. [But] right now, I think we reach the stage that anything you try should be in a clinical study, which forces you to adhere to certain standards, quality standards. (junior researcher, industry)

By “clinical studies”, the researcher is pointing to the need for interventional studies in the form of RCTs that would document the difference in patient outcomes for patients receiving PDO-based treatments, compared to standard procedures for treatment selection.

This call for statistical evidence may be somewhat surprising, as PDOs are often highlighted as personalized models intended to obviate the current reliance on RCTs. But to do this job, the method must first be validated via statistical evidence on patient populations, i.e., via evidence for which they are hoped to overcome the need for. As expressed by a cancer researcher in our study:

I think to implement patient-derived cancer organoids in the clinic, we, at present, would need a clinical trial. […] For that it needs a more, you know, concerted effort between clinicians, oncologists, surgeons, whatever, to put together a clinical trial, with the laboratory scientists, to see if there is an impact, game changing, in clinical management of oncology patients. (senior researcher, academic, cancer)

**Fig. 2:**
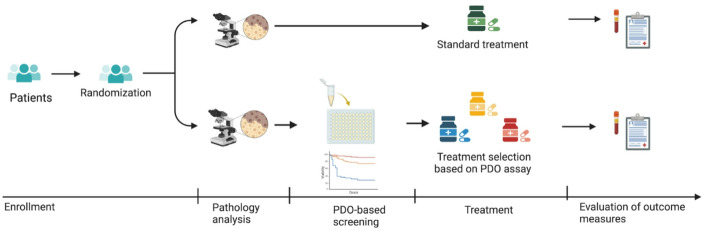
Illustration of the design of an RCT to test the clinical benefits of PDO-guided treatment selection. The figure is designed by the authors via BioRender.

The researcher elaborated that what is ideally needed to “plug the organoid into practice” (senior researcher, academic, cancer) is an RCT with two arms, one in which patients receive treatments suggested by their corresponding PDOs and another (control) group receiving the treatments oncologists would suggest without the organoid model. We illustrate the envisioned approach in Fig. 2, which is also inspired by the research protocols of the first registered interventionist phase III trials using organoids directly in patient management.^9^

In the interventional trial design, a group of cancer patients would be randomly assigned into the PDO-guided group or to a “control” arm, where treatment decisions are based on existing guidelines. This comparison is intended to provide evidence on the clinical utility of model-guided treatment selection, measured e.g. in terms of disease status, disease progression, and/or survival between the two groups over months or years. While this approach relies on statistical evidence, this situation is special as the purpose is not to evaluate the efficacy of a specific drug but rather how well “each model relates to each target patient” (Walker et al., 2019, p. 117). In other words, the RCT design is a set of n-of-1 trials exploring how well, statistically for the patient group, PDO-guided treatment selection can improve patient outcomes.

Although the field is still at an early stage, it is useful to engage in discussions about what it would take to validate PDOs for implementation in routine clinical care. Will PDOs obviate the need for RCTs or are they (still) considered necessary? How large should they be? Evidence from published phase III trials based on PDOs are generally lacking (Hofmann et al., 2023), although an analysis of registered clinical trials in international trial databases revealed that larger phase II and phase III trials are in the pipeline and that several are currently ongoing or recruiting (Vogt, 2025). Published results from interventional studies are currently limited to case reports for CF, and phase-I clinical studies^10^ and a single phase-III trial for cancer. We comment further on results from the most important initial studies below, as they are relevant for contextualizing what our informants saw as the pressing barriers for implementation. When asked about what it takes to validate the method of using PDOs for clinical decision making, our informants conditioned their responses with reference to specific contexts, often by contrasting CF and cancer. We unpack their responses in the following subsections stressing contextual factors such as available patients and their clinical situation, the time needed to meet clinical treatment needs, and the complexity of the disease itself.

### Available patients and their clinical situation

The informants in our study often clarified the need for PDOs, and evidence standards for their validation, by specifying contextual factors such as the number of patients available for possible clinical trials and their clinical situation. As mentioned previously, the small number of CF patients with specific mutational profiles was the very reason researchers started looking into PDOs as an opportunity for alternatives to RCTs as evidence:

It would be extremely hard to do [RCT] trials, to actually show statistically that the drug would also work for a patient with a particular other mutation. So, it is the same gene that can be hit in different places. So essentially, companies could not afford to do these trials, could not find the patients. You cannot do statistics on three patients. (senior researcher, industry)

The researcher reported that approximately 10 years ago, clinical implementation was initiated relatively quickly on an experimental basis in the case of CF. The speed of implementation was partly driven by demands of insurance companies that would not reimburse a newly available - but expensive - treatment without further proof of efficacy. Important was also that CF patients non-responding to standard treatment did not have other options:

[F]or cystic fibrosis it all went very fast because there was one drug, and this is a terrible disease, so the patient would get the drug or get nothing. (senior researcher, industry)

Before then, and parallel to the early implementation, observational trials had documented the correlation between the response of PDOs in vitro to drug cocktails and clinical indicators (Dekkers et al., 2016, Berkers et al., 2019, Ramalho et al., 2021). The largest observational, retrospective trial screened PDOs and clinical data from 173 CF patients and concluded that the FIS assay in vitro was highly relevant as predictor of what happens in the clinic (Muilwijk, 2022). Notably, some of these observational trials include elements of intervention. This is the case when researchers identify early in the study that some patients involved could benefit from a certain treatment that they are not currently benefiting from, and a decision is reached with physicians to prescribe that treatment. N-of-1, i.e., intentionally interventional trials with single patients have also been reported (Arora et al., 2021, Aalbers et al., 2022).^11^ Further steps have been taken towards standardization and qualification by health authorities with ongoing clinical trials to establish the validity of a centralized procedure that would collect cells from patients and grow PDOs to provide personalized guidance for treatment. Results documenting feasibility and safety (phase I) have recently been published (Bierlaagh et al. 2024).

CF is thus an interesting example of how PDOs already give some patients access to new drugs, without the background of published RCTs. This illustrates how evidence standards in practice can be modified through practical needs and clinical urgencies. However, several informants contrasted this to the context of cancer:

In the case of cystic fibrosis, we didn’t have to [rely on evidence from RCTs], because essentially it is either that drug or nothing. And there everybody, the patients, the doctors, were all very eager to give this a try. For cancer treatment, you know, there is always a treatment, and some of them are pretty good. So, you can only do this in the context of a clinical trial that is already happening, and you want to show that you are able to predict better what has been already done previously, that’s currently the case. And I think this step by step might be done in a phase 3 trial, which is bigger and more expensive. And eventually this is probably how it will find its way into the real clinical practice. (senior researcher, industry)

The researcher here emphasizes that in the case of oncology, the clinician cannot ignore existing treatment guidelines to try out PDO-guided treatments, as they are obliged to first try existing treatment options. Only when standard treatments no longer work do guidelines allow oncologists to explore opportunities for other treatments. This is also why most interventional trials using PDOs have thus far primarily been phase I trials in which patients have exhausted other treatment options (Vogt et al., 2025). The advanced and treatment-resistant status of the cancers of this patient group, however, constrains the number of patients who can be included in a trial and time window for researchers to grow organoids and produce clinically relevant test results (Green et al., 2022; Dam & Green, 2022). As outlined below, this temporal challenge is also different for cancer and CF.

### A race against time? The speed of organoid testing in CF and cancer

In the case of oncology, patients should receive treatments as fast as possible. They typically first receive a regimen of standard treatment(s) and then, in the case of nonresponse, perhaps a novel treatment or treatment-combination based on PDO-based drug testing. As the available patient group for PDO-based testing typically has advanced cancer, developing organoids for “real-time” clinical decision-making is challenging:

If you want to do organoids for patients in the context of their clinical path, then you’re dealing with something which is very different [from research]. You get a very small quantity of tumor cells because you start from biopsies, because usually these patients are not eligible for surgery, so you only get a small piece of biopsy. And then you need also to develop the best protocol to grow the material as fast as possible because you do not want to leave the patients without treatment for too long. (researcher, academic, cancer)

Developing enough material for a PDO test takes weeks or months, which is typically longer than cancer patients can wait for a treatment decision. In the words of a researcher from industry:

But establishing the organoids, it takes time. Time that we don’t have when cancer is already diagnosed. Usually, the cancer is in a more advanced stage, if you are able, or if organoids are indicated, so it becomes a race against time. (senior researcher, industry)

Moreover, the success rates for the establishment and growth of organoids vary between cancer types and even between patients with the same diagnosis. This challenge is experienced as very frustrating and heartbreaking not only to oncologists but also to the researchers:

I can tell you, personally, that was extremely frustrating and sad. I mean, we knew the patients and we wanted them to do well. And, unfortunately, we would get a response, and it was a very good response and led to a lot of insights, but after the patient died. So, obviously we want to move things quicker, and also make it more minimal so every patient can be tested and can have organoids used. (senior researcher, academic, cancer)

Thus, PDO-based screening in this context is like “running against the clock” (senior researcher, industry). Our informants’ experiences resonate with observations in previous ethnographic studies showing how both researchers and oncologists can feel stretched between the hopeful expectations of the PDOs and persistent uncertainties and practical challenges of growing organoids for drug testing fast enough to meet patient needs (Green et al., 2022; Dam & Green, 2022). This temporal challenge also spills over as an evidence conundrum:

What is difficult is that once we get the report, because it’s a phase 1–2 trial, the patient may have died already. So, it’s not a bias due to organoids, it’s really a bias due to phase one, because you have patients who have really advanced disease. (senior researcher, academic, cancer)

In translational cancer research, testing the benefits of PDO-guided treatment decisions is thus dependent on access to specific patient populations, which Dam et al. (2022) call “precision patients”. “Precision patients” must have sufficiently advanced and treatment-resistant cancers and stable disease status to fulfill the regulatory access criteria for experimental trial participation. But while the specific situation of “precision patients” opens a potentially mutually beneficial avenue for organoid research and access to treatments for patients, this also makes it difficult to validate the PDO method through this specific patient group.

The challenge described is also illustrated in the two largest prospective interventional trials on PDOs to date. One notable study is a phase-I trial using PDOs to guide treatment selection for patients with metastatic colorectal cancer (Ooft et al., 2021).^12^ Among the 61 enrolled patients, only 6 patients received PDO-guided treatments, either because of difficulties establishing PDOs or because the patients deteriorated before the result of the PDO-test could be used for treatment selection. For the 6 patients who did receive PDO-guided treatment, “patients did not demonstrate objective clinical responses to the recommended treatments” (ibid. p. 1). Another important publication is the first published phase III trial, a multicenter randomized study involving 137 patients with pancreatic cancer (Sarno et al., 2025). 44 patients were randomized into a conventional treatment arm and 81 to a precision medicine arm, where treatments targeting specific mutations were tested on both PDOs and “avatar mouse models”^13^ to guide treatment decisions. The result of the study showed benefits for a very small subset of patients (4 individuals) who received precision treatments, as the median overall survival was 19.3 months (compared to 8.7 months). Yet, the authors conclude that the FPM approach statistically “did not improve survival as compared with standard of care in an intention-to-treat population”, as “most patients could not receive a matched therapy because of premature clinical deterioration, delays in obtaining study results, or absence of actionable targets” (Sarno et al., 2025, p. 279). As the authors note, the background for these disappointing results is not necessary a failure of PDO-models as such. The difficulties stem from several combined challenges. First, not all cancer mutations can currently be considered “actionable” in the sense that available targeted treatments exist for these. Second, the success rate for organoid establishment and growth can vary between patients and is highly dependent on accessibility of tumor cells (which can be limited for advanced cancer patients with metastatic cancer). Third, the clinically available population of cancer patients for this type of translational trial often do not have time to wait for researchers to develop PDOs from such limited material (see also Dam & Green, 2022; Green et al., 2021, 2022).^14^

In comparison, the situation is remarkably different in the context of CF:

For CF, we have a progressive disease, people nowadays die in their 30–40s. If it takes half a year to get an organoid in your lab, to grow them, to make measurements with specific types of drugs, and after half a year you can feedback data and tell you about drugs, then you’d rather do it quicker, but half a year is still OK. If you look at cancer, it’s a completely different story. (senior researcher, academic, cystic fibrosis)

This researcher added that in the context of cancer, there is the further challenge that the mutational composition of tumor cells can evolve within months. A PDO based on a primary tumor is not necessarily a reliable “avatar” for future treatment testing, as the organoid – like the patient’s tumor – changes in response to environmental changes, including treatment exposure. Thus, for tumor organoids, the temporal challenge is not only to grow organoids fast enough for clinical needs but also in a pace where treatments are based on models that still mimic the patient’s cancer. This underscores how cancer – unlike CF – is a moving target that is genetically unstable and evolves over time (Green et al., 2022).

### Disease complexity and type of treatment response

An additional concern for cancer researchers is that a needle biopsy may provide insights into only a small part of the mutational dynamics of a patient’s tumor, as it is sometimes the case that organoids based on different biopsies in the same patients grow differently or yield different results. In the field, this is referred to as the challenge of *intratumor heterogeneity* and was contrasted to the case of CF as a monogenic disease in our interviews:

Within a cancer you have different types of mutations, all playing a part, and probably 1% of all the cells in a cancer are the ones responsible for metastasizing and ultimately killing the patient. Whereas if you go to a monogenetic disease setting, like CF, we have a stable background, we can do bulk measurements on the individual. …We don’t care about individual variations of organoids, we just do bulk measurements, because in a monogenic disease the entire tissue is affected in a similar manner - you just look at your total tissue response of the organoid. (senior researcher, academic, cystic fibrosis)

CF is a special case because the malfunctioning CFTR gene is detectable in cells beyond the organs where patients experience symptoms. As another informant (senior researcher, industry) explained, CF was traditionally viewed as a pulmonary disease, then considered an inflammatory disease affecting the pancreas and liver, and it is now considered a genetic condition with mutational variations detectable also in cells from rectal biopsies (see also Dekkers et al., 2016; Berkers et al., 2019; de Poel et al., 2020). Intestinal organoids currently have a much higher success rate for growth in vitro than tumor organoids; one researcher estimated a success rate of 95% from rectal biopsies for CF, compared to 30–40% on average for tumor organoids (senior researcher, academic, cystic fibrosis). Although this difference may be partly explained by the expertise developed in central laboratories working on intestinal organoids also for other purposes, establishing and growing diverse cancer cells as tumor organoids is more challenging. This currently leads to unpredictable success rates and high variation between the organoids of different cancer types and patients.^15^

The organoid readout also differs for CF and cancer. In the case of CF, a malfunctioning CFTR gene is detectable by cells “failing” the previously mentioned swelling test, as the cells do not allow liquids to enter (via the CFTR channel). In the case of cancer, the readout is instead cell death or reduced cell viability, which is a test result that can be difficult to distinguish from confounding factors such as suboptimal growth conditions or differences in the handling of treated and untreated (control) organoids:

For CF it’s very simple, basic[ally] it’s fluid secretion or ion transport. And for cancer, I think it’s difficult, either cytotoxicity or just LD50 doses or so, or potentially selective killing of cancer cells versus normal cells, this type of things. So, I think in general there needs to be a lot of validation being done still, and validation for me means direct measurements of in vitro and in vivo data. (senior researcher, academic, cystic fibrosis)

Thus, the PDO model readout can be harder to interpret in the case of cancer, compared to CF. A similar difference is seen in the measurement of treatment responses in patients. An informant explained that for patients with CF, who do not respond well to standard treatment, access to new drugs via PDOs can be “essentially lifesaving, with the drug you become a normal person” (senior researcher, industry). In the case of targeted cancer treatments, in contrast, successful outcome measures often amount to months of progression-free survival, which is difficult to evaluate at the individual level (see also Vogt & Hofmann, 2022). Thus, the barrier for clinical implementation of PDO-based treatment screening is dependent both on the complexity of the disease studied and the type and degree of performance of targeted drug therapies. These differences, in turn, impact the sample size needed for validation. On this basis, we return to the discussion of the relationship between evidence in EBM and FPM.

## Revisiting evidence standards in precision medicine

PM might change what “evidence-based medicine” will mean in the future. From an optimistic perspective, limitations in statistical trial power may be counterbalanced by larger effect sizes of precision treatments and a greater mechanistic understanding of diseases (Tonelli & Shirts, 2017; Andreoletti, 2018). However, as mentioned in the introduction, the effect sizes of precision treatments are often insufficient to counterbalance other limitations in trial power (Plutynski, 2022; Tabery, 2023), and mechanistic reasoning is often insufficient for making robust inferences about what works in an individual (Vogt & Hoffman, 2022; Vogt, 2025). Against this backdrop, FPM is philosophically interesting as a new approach for addressing this problem via *functional* models aiming to document predictive performance over time in patient-specific models (Letai, 2017; 2022; Bose et al., 2021).

As Walker et al. (2019) have argued, inferences about patient response from PDO-based testing are not directly grounded in mechanistic evidence, as the causal mechanisms influencing drug response are often unknown and black boxed in the testing phase. Indeed, our informants also emphasized that for this purpose, they do not need to understand *why* a treatment works but only *that* it works.^16^ Just as the utility of the antibiogram test does not rely on correct classification of the pathogen, PDOs can be valuable as diagnostic tools for treatment allocation, independent of whether researchers understand the full causal basis for the treatment effect. However, PDO-based testing rests on a mechanistic rationale in the sense that essential aspects of treatment response are taken to be represented in the PDO, as both CF and cancer are here taken to be genetically based diseases. Moreover, the informants’ reflections about uncertainties in the two cases also illustrate how insights into disease mechanisms are relevant as background for the scientists’ views on how well a PDO can represent relevant aspects of the studied disease. While CF is a monogenic disease with a well understood mechanism affecting how cells regulate their salt-water balance, “cancer” refers to multiple distinct diseases, each involving multiple genes that create complex cellular dynamics that also evolve over time.

Insights into the complexity of the diseases studied also influence the informants’ views on the requirements for model validation. Although the PDOs are based on patient tissue or cells, their validity as predictors of patient response cannot be taken for granted. A senior researcher explained that the data from both laboratories and clinics can be “very noisy at the individual level”. Hence, an RCT such as the design in Fig. 2 was considered needed. But the informants also emphasized that what constitutes a proper trial and trial size depends on the context. One researcher explained that in the case of CF, having a sample size of only 20 patients can provide an idea about the relative spread of variation between the in vitro model and the in vivo target (senior researcher, academic, cystic fibrosis). Variation here refers to clinical variation in symptoms and treatment responses, correlated with different mutations in the CFTR gene.^17^ Larger collaborative trials are in the pipeline to explore the variation in mutations, PDO results, and patient responses among several hundred CF patients, as a basis for power calculations of future trial sizes. Our informants generally contended that the required sample size will be lower for CF than for cancer, because the former is less heterogeneous:

For CF at least we start to see good correlations from these types of numbers [20]. But we have a disease where it’s a bit more potentially simple, for cancer you need bigger numbers. The more the better. (senior researcher, academic, cystic fibrosis)

A cancer researcher similarly explained that even a relatively large planned RCT with 300 patients might not provide a sufficient patient sample to account for differences between the PDO-based and the control group. One challenge is that the population of cancer patients is generally more diverse than that of CF patients, as they have different mutations and mutational combinations in multiple genes. This makes it difficult to draw inferences about differences between the control- and intervention groups without large sample sizes. Moreover, unlike CF, PDO-testing can involve selection among available standard treatments, genomically indicated targeted treatments, *or* genomically indicated off-label treatments (i.e., treatments approved for other cancer types). These factors complicate the combinatorial matrix of comparison in a trial:

The goal is to determine whether the control arm that is not treated based on organoids do better or not than the arm that has been treated with the organoid oriented treatment. So, you can randomize. However, it’s 300 patients, and to do that you need a much larger reservoir of patients with the same indication. [I]n the panel we are testing on, organoids will have drugs that are given in digestive cancer, but we have also drugs that are given in other indications such as breast cancer, lung cancer and others. (senior researcher, academic, cancer)

As a result of this complexity in the context of cancer, larger numbers are therefore needed for the ideal trial to evaluate the clinical utility of PDOs in cancer medicine. It is somewhat paradoxical that the evidence barrier for precision medicine is the lack of large patient samples, as precision medicine is being highlighted as a departure from traditional evidence-based medicine and the reliance on aggregated and large numbers. However, as our analysis shows, researchers in precision medicine grapple with the persistent difficulty of evaluating what works in an individual. To validate the candidate “personal models”, the field still must rely on statistical evidence, especially when disease complexity is high or when expected effect sizes of targeted treatments are moderate or uncertain.

## Discussion and concluding remarks

Precision medicine is motivated by the insight that the existence of specific genetic mutations can impact disease development and treatment response. Stratifying patient populations according to genetic variants, however, comes with the evidence challenge that patient groups with the same disease decrease - in extreme cases down to a few or even single cases. We have illustrated this issue through the cases of cancer and cystic fibrosis. The latter– despite its prevalence as a monogenic disease – is caused by diverse mutational patterns and symptoms, which can require different treatments. Similarly, with PM, cancer is increasingly referred to as a multiplicity of different diseases because of the plethora of mutational variants. Hence, PM may lead to redefining traditional disease categories into numerous subcategories or rare diseases (Wadmann & Hauge, 2021), which has implications for what constitutes evidence.

PM is often conceptualized as a departure from the reliance on statistical evidence in classical EBM, enabled by predictive biomarkers and a greater molecular understanding of diseases. But while PM promises to provide a better evidence basis for treatment decisions, it also – paradoxically – comes with new forms of uncertainty. A comment in *Nature Reviews Clinical Oncology* argues that PM is characterized by a fundamental paradox: as efforts to account for unique features of individual patients intensify to reduce uncertainty in the clinical management of patients, PM becomes increasingly dependent on low-grade evidence, such as studies with small patient populations and reliance on case reports (Kimmelman & Tannock, 2018: see also Vogt & Hofmann, 2022). Proponents hope that the limitations to trial power, following increasing patient stratification, can be outweighed by a more comprehensive mechanistic understanding of diseases (Tonelli & Shirts, 2017; Andreoletti, 2018). However, a fundamental gap persists between the increasing availability of molecular data and the ability to predict what works in an individual (Vogt, 2025). While targeted treatments are becoming available, it remains a challenge to predict which patients will benefit from these.

We have explored what constitutes evidence in the context of the emerging field of functional precision medicine (FPM), which addresses the gap between molecular information and functional testing. FPM has here been exemplified through an analysis of the use of PDOs to guide medical decisions at the individual patient level in the context of CF and cancer. Although it seems intuitive that organoids based on the same person’s cells are reliable indicators of treatment response, PDOs do not straightforwardly represent the patient’s disease. As stressed in our interviews, scientists in the field are well aware of epistemic uncertainties and acknowledge the need for model validation and documentation of clinical benefits. While PDOs for CF and some cancer types have presented positive predictive validity in prospective observational trials, assessment of the clinical utility requires further documentation that PDO-guided treatment decisions statistically improve patient outcomes, compared with existing guidelines for treatment selection. Hence, in the views of our informants, arriving at reliable personal models requires population-based approaches.

Our analysis further reflects how the perceived evidence requirements can be context-dependent (see also Fuller et al., 2024). As this is an exploratory analysis at the early stages of implementation, it is hard to know whether the identified tendencies generalize. But differences highlighted in existing scientific literature were emphasized by our informants, who often contrasted the current situation in CF and cancer medicine. Cancer is considered more mechanistically complex than CF, the “signal” in PDO-based testing is often less clear, and the effect sizes of targeted cancer treatments are harder to evaluate at the individual level. As a result, the sample size required to validate “personalized models” is considered much larger in the case of cancer. Our informants also clarified that the evidence requirements depend on the specific clinical situation of patients. The implementation of PDO-based screening was faster in the case of CF, because the patients enrolled in the early experimental trials did not have other treatment options, and PDOs allowed some patients to access a new and expensive drug. This is unlike most cases in oncology, where current guidelines require oncologists to first try several different indicated treatments. This situation generates an evidence conundrum for organoid researchers in cancer medicine, as the “precision patients” (Dam et al., 2022) available for clinical trials typically have advanced cancer and limited time to wait for their PDOs to grow to a sufficient size for drug testing. Moreover, the number of potential targeted (experimental) treatments is greater for cancer, which adds to the complexity of designing RCTs to document the efficacy of alternative treatment options.

Researchers hope that improvements in laboratory protocols and the validation of PDOs in larger phase III trials can overcome existing hurdles. However, when asked about the short-term and long-term prospects of PDOs for clinical application, several informants also highlighted that it remains to be seen for which purposes they are realistic to implement. For example, a senior cancer researcher said that the PDO-approach may not work for everybody or be practically feasible for every hospital. Others stressed that challenges to grow tumor organoids for (some) advanced cancer patients may be very difficult to overcome. These perspectives are also supported by the previously cited phase III trial on pancreatic cancer (Sarno et al., 2025). Rather than being a revolutionizing tool for individualized prediction in every field, the clinical utility of PDOs is currently highly context dependent. Moreover, it remains uncertain whether all diseases (or even all cancers) can be - and should be - modeled in vitro and at the individual level (see also Green et al., 2021, 2022). Regardless of such fundamental challenges in some contexts, FPM deserves more philosophical attention, and we hope that our analysis contributes to a first step in this direction.

FPM presents an interesting alternative pathway that neither requires a detailed bottom-up understanding of genotype-phenotype relationships, nor rejects the importance of statistical evidence in medicine. The comparison of PDOs to individualized antibiograms illustrates how functional testing above the molecular level may pave the way for a “radical personalized medicine” (Fuller, 2025, p. 410), without requiring a radically new epistemology. First, while relying on mechanistic assumptions about genetic variation strongly impacting treatment outcomes, PDOs can function as an “epistemic shortcut” (Walker et al., 2019) that allows researchers and clinicians to ignore some of the complexity of molecular interactions. Second, as our analysis shows, there is a continued need for validation of PDO-models for clinical use though statistical approaches such as RCTs. Developing personalized models thus paradoxically requires the use of the similar evidence-practices as PM is hoping to overcome the need for. Importantly, however, in this context the RCTs are not intended to document the efficacy of a specific treatment in a population. Instead, an alternative RCT design is suggested to assess, through a series of n-of-1 studies, whether PDO-guided treatment decisions can significantly improve individual patient outcomes. In this sense, FPM is not an antagonist to EBM but rather an approach that relies on both functional testing and statistical evidence to compare patients in novel ways.

## Footnotes

1 It can be debated what defines EBM, historically and today, including the extent to which EBM privileges RCTs and meta-analysis (Zuiderent-Jerak, 2021; Fuller, 2025). In this paper, we are primarily concerned with how proponents of PM view EBM and what is presented as a better approach.

2 https://www.sfpm.io/, accessed Nov 11, 2024.

3 Organoids are also used as preclinical models for research and drug development. We focus here on clinical application with direct involvement of patients to extract the features most relevant for PM.

4 We invited approximately 20 scientists in total to reach 10 accepted interview invitations. 7 informants were affiliated to a European institution at the time of the interview, 2 from Asia, and 1 from the USA. 4 interviewees had a connection with the industry but some of them retain academic affiliations as well. Most informants were focusing on different subtypes of cancer and 2 had experience in CF research. 9 of them had more than 10 years of experience in research after their PhD (“senior researchers”). Some elements of context are provided in parenthesis after each quote (e.g., senior/junior, area of expertise, main affiliation in academia or industry). However, due to dual affiliations and recent career changes (e.g., geographic relocation or shift from the academic sector to the industry), this kind of categorization is, in the end, not that relevant for our analysis.

5 Forskolin is a natural diterpenoid compound commonly used in laboratory research to stimulate enzymatic production of cyclic AMP (cAMP), which is involved in a range of intra- and extracellular signaling pathways.

6 We thank Sophia Efstathiou for comments on this.

7 Also here, there is a parallel to visions in clinical microbiology; the use of omics information and machine learning has been suggested to develop “personalized antibiograms”, thus using antibiograms as links between bacterial genomic biomarkers and for “precision antibiotic prescribing” (Corbin et al., 2022).

8 The trial is “co-clinical” or “parallel” trials because the model is not actually used as a guide for prognosis and drug prescription as a typical clinical trial experimenting with a new treatment (or repositioning a treatment validated for other conditions).

9 An example is a planned RCT at Helsinki University hospital, which is registered with trial ID, NCT04842006 (clinicaltrial.gov) and is recruiting patients with colorectal cancer. We identified only four additional active RCTs in the trial database, all in China. We comment on the first published RCT later in this paper.

10 In phase-I studies, PDOs are used to screen for alternative (off-label) treatment options for patients who do not respond to standard treatment.

11 These reports document the strategy of using PDOs in the clinical management of patients. There are probably many more CF patients for whom the functional test has authorized a drug on compassionate use, but we do not have access to a complete record of all patients who have been treated through this strategy.

12 We only identified a few similar trials, often without resulting publications. Our informants hinted to lacking publications from registered phase I trials as a reflection of similar challenges in other studies (see also Green et al., 2022).

13 Mouse avatar models refer to patient-specific mouse models xenografted with tumor tissues of specific cancer patients. For a philosophical discussion of such models, see (Green et al., 2021).

14 Other research teams are currently exploring the capacity of organoids to personalize standard treatment decisions also for newly diagnosed patients, based on PDO-testing of chemotherapy response (Schmäche et al., 2024). Discussion of the implications of this application is, however, beyond the scope of this paper and will be explored in a future publication.

15 Adding to the challenges is also that tumor organoids tend to die if the cell culture becomes too dense. To grow optimally, they need to be divided up and placed in new wells with fresh growth media. This involves labor-intensive work that can introduce errors and variation in samples. Some of these challenges may be overcome with improved (and automated) laboratory procedures, but there may be inherent biological limits to how much the speed of cell growth can improve.

16 For a more general discussion of the role of mechanistic evidence versus statistical inferences, see (Stegenga, 2018a, 2022).

17 Some researchers may for instance measure a treatment response in the lungs of one patient, whereas another patient may have a measurable response in tests of pancreatic function, and it is then unclear how to compare and quantify these.
